# Development and Introduction of the Filariasis Test Strip: A New Diagnostic Test for the Global Program to Eliminate Lymphatic Filariasis

**DOI:** 10.4269/ajtmh.21-0990

**Published:** 2022-03-15

**Authors:** Anastasia Pantelias, Jonathan D. King, Patrick Lammie, Gary J. Weil

**Affiliations:** ^1^ Bridges to Development, Seattle, Washington;; ^2^ World Health Organization, Geneva, Switzerland;; ^3^ Centers for Disease Control and Prevention, Atlanta, Georgia;; ^4^ Washington University, St. Louis, Missouri

## Abstract

A key component to achieving the global goal of elimination of lymphatic filariasis (LF) is the availability of appropriate tools for disease mapping, monitoring, and surveillance. However, the development of these tools for a neglected disease such as LF can be a challenge. The lack of a commercial market and low familiarity with these diseases leave little incentive for diagnostic manufacturers to invest in this space. The Filarial Test Strip (FTS) development story provides a case study on how a multi-stakeholder, public–private partnership model facilitated the development, evaluation, and introduction of a new monitoring and surveillance tool for LF. This paper will reflect on the experience with the FTS and document the process from development of the target product profile to adoption and scale-up in country programs. Lessons learned from both the successes and challenges experienced during this process may help inform future efforts to develop and introduce new diagnostic or surveillance tools for neglected diseases.

## INTRODUCTION

Lymphatic filariasis (LF) is a parasitic disease that is a major cause of chronic disability in the developing world. According to the 2021–2030 road map for neglected tropical diseases (NTDs) published by the World Health Organization (WHO), the global goal for LF is elimination as a public health problem by 2030 through repeated rounds of mass drug administration (MDA). ^1^ Critical components of any elimination program are monitoring and surveillance. Appropriate assessment tools and methods are needed for each stage of an elimination program; mapping to identify which areas require intervention, monitoring to assess the impact of interventions, and post-intervention surveillance to validate elimination or detect recrudescence. ^2^^,^ ^3^

About 90% of the world’s LF cases are caused by *Wuchereria bancrofti*. ^4^ Available methods for detecting active infection with *W. bancrofti* include detection of microfilariae (mf) by microscopy on blood drawn typically at night (based on the periodicity of the parasite), detection of circulating filarial antigen (CFA) in blood, and detection of filarial DNA through molecular methods such as polymerase chain reaction (PCR). ^3^ Currently, the most widely used method is detection of CFA in blood because of its combination of sensitivity, specificity, and ease-of-use, as it can be performed using blood samples from any time of the day. The first CFA antigen tests were laboratory-based, antigen-capture assays such as enzyme-linked immunosorbent assays (ELISA) or radioimmune assays. However, in the 1990s, a commercially available, point-of-care (POC), lateral flow assay (LFA) was developed and offered by the Australian diagnostic manufacturing company ICT Diagnostics. This test allowed programs to conduct infection assessment surveys entirely in the field; no longer reliant on either night-blood-draws or laboratory assays. ^5^^,^ ^6^

### The problem.

Over time, this test has been fully integrated into protocols for LF elimination programs including those for endemicity mapping, sentinel and spot-check monitoring, MDA stopping decisions, and post-MDA surveillance, making it an essential tool for the success of the Global Program to Eliminate Lymphatic Filariasis (GPELF). ^7^ In the 2000s, the test was acquired and technology transferred to the U.S.-based company, Binax, Inc. Binax continued to manufacture and offer the test, the BinaxNOW Filariasis card test, even after being acquired by Alere. However, during the course of these technology transfers, LF programs noticed decreases in test sensitivity, test result stability, product shelf-life, stability, and variable performance. ^8^^,^ ^9^ In addition, the high cost of the test and specific technical issues created challenges for the program. Technical challenges included a short shelf life of only 3 months at ambient temperature or the need for a cold chain to extend shelf life, an impracticality in most areas where the test is used. In addition, performance of the test mandated a very narrow read-window of 10 minutes and reading the test too late often resulted in false positives. ^10^

Because of the criticality of this test for the GPELF, the challenges described above posed problems for implementing partners, and led to a multi-stakeholder meeting to discuss the issues and engage the manufacturer as a partner to help solve them. The situation presented an opportunity for a unique public–private collaboration that enabled the development, field testing, and eventual access to a new and improved CFA antigen test for LF. ^11^^–^ ^13^ Funded by the Bill & Melinda Gates Foundation, the collaboration included the diagnostic manufacturer (at the time, Alere, Scarborough, ME) that was responsible for developing the next-generation test; the LF community that was responsible for defining the target product profile (TPP) and conducting field evaluations of the new test; the WHO, which agreed to manage centralized, batched procurement of the new test; and the drug-donating pharmaceutical companies who agreed to fund the procurement of those tests. The result of this effort was the development and introduction of the Filariasis Test Strip (FTS).

The LF community’s experience with stimulating development of the FTS is significant because it represents the successful introduction of a new tool for NTDs despite the lack of commercial market and low incentive for private industry partners because of low volumes and very tight margins. In addition, while working directly with the commercial partner expedited the development process, the introduction and scale-up of the new FTS and phase out of the BinaxNOW card test was more complicated and, therefore, took longer than anticipated. In this review, we will look back on the history of the FTS, the successes, and remaining challenges, and explore how lessons learned from this experience may be used to inform the introduction of other new next-generation surveillance tools for NTDs.

### Public–private partnership for development and introduction of the FTS.

#### Product development.

The experience with the FTS highlights how both public and private partners can come together to support the rapid development and introduction of a new tool for global health. Alere (now Abbott), the manufacturer of the BinaxNOW filariasis card test at this time, was willing to redesign the test to address the challenges expressed by the GPELF. However, Alere’s involvement in LF was historical. Alere acquired the BinaxNOW card test during the acquisition of Binax, a diagnostic manufacturer based in Scarborough, ME. Binax had a minority stake in the Australian company, AMRAD-ICT, and after AMRAD-ICT shuttered, the card test technology was transferred to Binax, Inc., in Maine (now a part of Abbott). Because of the technology transfer and multiple acquisitions, at the time of the test redesign, Alere lacked deep institutional knowledge of the test and of LF. Therefore, to meaningfully engage in this project to improve LF surveillance tools, Alere would require a detailed TPP, which was developed in 2009 through a series of workshops by members of the GPELF. Key aspects of the TPP included a lower cost per test, improved temperature stability, and a longer read window with improved stability of test results. These improvements would make the test more affordable for programs and address the key logistical and end-user challenges associated with the BinaxNOW card test.

In 2010, the process to develop the next-generation LF test was initiated, funded in part internally by Alere and, in part, externally by the Bill & Melinda Gates Foundation. At first, the donor and manufacturer assumed that it would be sufficient to make minor modifications to the existing card test; however, it quickly became apparent that to meet the TPP, particularly the cost target, the manufacturer would need to fully redesign the test. Because the manufacturer was committed to the project, they dedicated both human and financial resources toward this project that allowed for the development of a prototype FTS. In addition, this rapid timeline was facilitated by the engagement of GPELF. Organizations such as Washington University (St. Louis, MO) and the U.S. Centers for Disease Control (Atlanta, GA) conducted laboratory-based evaluations of prototype tests and provided both data on test performance ^12^ as well as feedback on user-experience and test design (form factor). Ultimately, consultation with GPELF provided Alere with the necessary insight into how LF programs work in the field, how the test would be used, and under what environmental conditions. The ability of GPELF to provide input to the diagnostic manufacturer during the development process enabled Alere to compress their timelines such that a product could be provided to GPELF within 1 year for field testing and further refinement.

#### Field testing and incorporating FTS into WHO guidelines.

Once the laboratory evaluations were completed, the next step was to move the test into the field to gather the evidence needed to include the FTS in the WHO’s monitoring and evaluation guidelines for LF. The first field studies were done in 2012 and involved parallel testing of the BinaxNOW ICT card and the new FTS, with microfilaria testing as the gold standard. The studies were designed to assess and compare sensitivity, specificity, and test result stability between the old and new tests. These studies took place in different settings in Africa and Asia in areas with and without prior MDA for LF. Results of these evaluations were presented at international meetings, shared with key stakeholders, and published. ^12^^,^ ^13^ In some cases, the field studies were embedded within already planned program activities such as mapping, sentinel site evaluations, and transmission assessment surveys (TAS), allowing for a more rapid transition from product development to field evaluation.

For WHO to consider whether the test was acceptable for use in GPELF, evidence of both laboratory performance and field performance, including usability, had to be generated. In August 2014, 2 years after the start of field testing, all available evidence from studies to date was reviewed during a WHO-convened meeting of a subgroup of the NTD Strategic and Technical Advisory Group. ^14^ The following minimum information from 15 field studies was considered: the protocol followed; the description of the sample population surveyed; quantitative results including the percentage of agreement between the FTS and the ICT, and the prevalence estimate or point estimate of antigenemia as determined by each test; qualitative results including the program decision indicated by survey results of each test; and operational observations and feedback from technicians. Overall agreement for individual test results between FTS and ICT ranged from 89.6% to 100%. The laboratory evaluation demonstrated that the FTS could detect lower concentrations of CFA than the ICT and this was felt to explain why, in all but three field studies, more people tested positive with FTS than ICT. ^12^ Despite this apparent increase in sensitivity, in all but a single study, the results led to the same programatic decision regardless of test used. ^14^

Feedback from the end-users in study reports was invaluable. More positives or invalid tests were reported on the first days of use in the studies incorporating the FTS in routine programatic surveys. This indicated the need for additional, intensified technician training prior to deployment of the diagnostic test in the field, leading WHO to develop with CDC support, new bench aids, and training videos to assist end-users. Several disadvantages of FTS compared with its ICT predecessor were reported, including flimsiness of the strip when compared with the card test format and, therefore, the need to secure the strip to something during use. As a result, the FTS was more difficult to use in the field where a small wind could displace the strips. ^14^ In addition, the strip lacked space to write a person’s unique identification number to ensure confidentiality and accuracy of recording results. Based on the feedback, WHO discussed potential solutions with the manufacturer who agreed to make minor changes such as adding a plastic U-shaped tray for the strip to sit within so that it is protected from wind and more stable in the field and to providing space for users to label tests using unique IDs.

#### Procurement and introduction.

With good partner coordination at the global level, field testing was soon completed and the FTS demonstrated lower cost and improved performance. The WHO and GPELF supported transition from the BinaxNOW card test to the new FTS and the test was integrated into existing guidelines and recommended for decision-making surveys within programs. This occurred within 5 years of the initial 2010 investment to support the development of an improved test. It was, however, wrongfully assumed that the transition would be smooth; just replacing the card test with the FTS in LF programs did not ensure rapid uptake by country LF programs. These programs were not familiar with the new FTS and they were reluctant to change to a diagnostic with different performance characteristics. Of particular concern was the increased sensitivity of the FTS, which might mean that programs would not reach their elimination goals as quickly as anticipated. The slow uptake by national programs and low volumes of tests required at the time presented a logistical challenge for the manufacturer. The low-cost design of the FTS was unique to its product line and required its own production run. With competition from other, much higher volume tests for infections such as influenza and HIV, Alere struggled to schedule production of low volume, one-off orders of the FTS, leading to long delays. In addition, the BinaxNOW card test was still available and being used by LF programs. A solution was needed to facilitate the transition to the FTS in country programs and streamline the ordering, production, and shipment process at Alere.

As a first step, Alere agreed that the BinaxNOW card test would be phased out of production, a move that would force the transition to the lower-cost, better-performing FTS, and simplify production and manufacturing demands on the company. Another mechanism to facilitate the transition was the development of an agreement by the drug-donating pharmaceutical companies and the Bill & Melinda Gates Foundation to provide resources for procurement of the new FTS. The three companies that donate the drugs needed for LF elimination—GlaxoSmithKline (GSK), Merck & Co., Inc., and Eisai—along with the Gates Foundation, completed a Memorandum of Understanding to provide resources for the purchase of the FTS by the GPELF for use in country programs. This was a unique agreement whereby drug-donating companies agreed to support purchase of the diagnostic tests needed to monitor and evaluate the programs they support through their drug donations and other contributions. It also represented an important gesture of good faith to Alere that resources would be available for the transition to the new test.

However, before these efforts to fully transition to the FTS could truly take effect, the logistics around ordering, production, and shipment needed to be streamlined. Accurate demand forecasts and consolidated ordering would allow Alere to plan manufacturing runs. This better coordinated and consolidated ordering and shipping process was enabled by the improved temperature stability and increased shelf-life of the FTS. World Health Organization agreed to take on the role of central procurer for the FTS. This coordination effort required financial support from the partners who agreed to support the purchase of the diagnostic and provide WHO with the resources to take on this role. Donors also agreed to provide resources to cover shipping and WHO facilitated customs clearance for the tests, and these steps led to improved uptake by programs.

World Health Organization incorporated the request for diagnostics in the existing reporting mechanisms and request procedures for donated medicines. To access the subsidized/free FTS, countries were requested to submit a letter of request and a report of the progress made to date indicating a country program’s eligibility to conduct the WHO-recommended survey in which the FTS would be used. These requests were reviewed by WHO and by Regional Program Review Groups where active. Given that the new test did not have regulatory approval in many LF-endemic countries, a government-signed “no objection” letter indicating that the test could be used in the country for the purpose of LF elimination programs facilitated importation.

WHO developed demand forecasts by reviewing progress of the countries with MDA and assumptions that national programs would follow the GPELF strategic framework and conduct surveys at the recommended time. Estimates were derived by the standard sample sizes used in TAS and prediction of the number of evaluation units eligible for conducting TAS. Since the inception of the global coordination mechanism, this forecasting has involved more supporting partners, donors, and WHO regional offices. Currently, monthly meetings are held with the manufacturer and the major FTS-procuring partners and partners providing financial support for survey implementation. These meetings help update all stakeholders on stock availability, forecasts, prioritization of orders, and serve as a mechanism to solve any logistics issues.

Since 2015, WHO has procured more than 3 million FTS on behalf of LF-endemic countries for recommended surveys. The current global demand for FTS for use in GPELF is approximately 1 million tests per year. USAID implementing partners procure FTS for LF-endemic countries that were supported by ENVISION and currently by ACT to End NTDs, which makes up about 40% of the global demand annually.

### Lessons learned from the FTS experience.

The experience with FTS can provide specific lessons related to the development, manufacturing, introduction, and scale-up of new or improved tools for NTDs. For example, this experience demonstrated *the value of a public–private partnership for product development of new public health tools with little to no commercial or private market* because it was driven by champions from the disease community, the commercial partner, and external donors. The partnership between the GPELF and Alere facilitated by the support of a willing donor, successfully brought together disease expertise with technical diagnostic development expertise to produce a new monitoring tool which met the performance and field-ready characteristics needed to support LF elimination efforts. In addition, the timeline from the development of the TPP by the GPELF to having a new test available for field studied was relatively rapid. Similarly, lessons can be learned from the approach to field evaluation of the FTS. These were done by nesting the evaluation within planned research and surveillance activities with an aligned donor. This enabled the GPELF to generate the evidence needed for a recommendation more efficiently and inexpensively without the need for separate, dedicated field validation studies. This cost- and time-savings approach should be considered in the future when evaluating improved surveillance tools for NTDs.

Another key lesson from the FTS experience is the need for *a positive relationship and open lines of communication among the disease community, the ultimate end-users of the product, and the commercial partner*. A key role that the Bill & Melinda Gates Foundation played during this process was to facilitate and bridge communications as a means to arrive at solutions that worked for all parties. Although the development, validation, and regulatory approval of FTS were accelerated, it became clear that just developing a tool, which meets the TPP, even when it was aligned to the global program, was not sufficient for it to realize its full potential. One needs to consider the logistics surrounding the production of and access to the product even before it becomes available. In this case, scale-up of the FTS was delayed because of challenges in logistics that were not considered until the tool was available. The established lines of communication were necessary to develop and implement solutions. In the future, however, delays can be avoided by planning for the downstream commercial introduction and access issues in parallel with the development and field evaluation process. World Health Organization has subsequently established a Manufacturing and Regulatory Pathways subgroup within the framework of its Diagnostic Technical Advisory Group, and these issues are an important part of that subgroup’s remit.

Finally, this work provided some *insight into the challenges of developing and introducing commercial products for diseases that suffer from a lack of commercial market and little to no commercial incentive*. For example, in the case of commercially available NTD diagnostics and surveillance tools, it is helpful to understand the business model of diagnostic manufacturers to better appreciate whether and how commercial partners could be incentivized to develop tools for NTDs. Diagnostic and surveillance tools for NTD programs often need to be very low cost, and they typically have low profit margins. This coupled with low volumes creates a significant risk to the long-term sustainability of these tools for commercial partners. In the FTS case, consolidating orders for the tests gave Alere the needed predictability for scheduling FTS production runs and the ability to produce a larger volume of tests at once, which is more cost-effective than producing tests in response to smaller volume, one-off orders. In addition, the fact that the new FTS had a longer shelf-life and no longer required a cold chain were key elements that enabled this arrangement. Still, these improvements do not remove the risk of de-prioritization in the frequently volatile world of diagnostic development and manufacture, where mergers and acquisitions leave neglected and rare disease diagnostics at risk. This risk is compounded when programs have to rely on a single manufacturer for a program-critical test. Finally, diagnostic programs cannot be equated to the drug donation programs for NTDs. Unlike drug donation programs, the FTS development program would probably not have been viable without external funding and guaranteed purchases by donors and WHO.

## CONCLUSION

Developing new surveillance or diagnostic tools for NTDs represents a challenge driven by the ultra-low cost required for procurement by public health programs, the low margins associated with these tools, and the lack of a commercial market that can help offset to cost of producing and offering these tools to the public health community. The development and introduction of the FTS was feasible because of the strong commitment of the donors and the manufacturer to this project. It was further advantaged by the fact that it did not require full *de novo* development. In other words, the FTS was a next-generation version of a well-accepted, established product produced by the same manufacturer using many of the same reagents. In addition, there was rapid commitment by the LF community to evaluate the test in the laboratory and in the field. Open communication with WHO on what evidence was needed for a recommendation also helped accelerate the process to move from field tests to introduction and scale-up. Finally, the commitment on the part of the drug-donating pharmaceutical partners to pool resources to procure the test facilitated the switch from the BinaxNOW card test to the new FTS and enabled scale-up across LF programs. Overall, the effort required the commitment, communication, and partnership of many actors across the private and public sectors to make the FTS story a success.

Despite the success, the FTS experience also brings up a few important considerations for the future. Although the GPELF was highly involved in the development of the TPP for the FTS, there was little end-user input into the design or form factor of the test. ^14^ Once the design was locked and the test was in field trials, additional changes to the design could not be made in response to feedback from country LF program staff that had used the test in the field. In the future, active solicitation of end-user feedback early in the design phase would ensure that a new surveillance tool not only would meet all the analytical specifications but also fit into the workflow and would be appropriate for the environments where the test will be used. The procurement mechanism established has worked only because of the external funding. Funding remains committed for WHO procurement of LF diagnostics through 2023. If the funding was no longer available, WHO could not sustain procurement of FTS on behalf of endemic countries. Finally, the FTS experience also raises the question of whether there are other business models to consider for NTD products that do not rely on the sustained commitment of a single commercial partner. For example, would bundling several NTD products make production of NTD diagnostics more attractive to manufacturers? Could this model also provide an opportunity for NTD donors to support a guaranteed supply of high-quality diagnostics? There may be value in considering and testing potential new pathways or models for overcoming the key challenges in developing new or improved diagnostic and surveillance tools for neglected diseases.
